# Cyanoethylation of the glucans dextran and pullulan: Substitution pattern and formation of nanostructures and entrapment of magnetic nanoparticles

**DOI:** 10.3762/bjoc.8.63

**Published:** 2012-04-13

**Authors:** Kathrin Fiege, Heinrich Lünsdorf, Sevil Atarijabarzadeh, Petra Mischnick

**Affiliations:** 1 Institute for Food Chemistry, Technische Universität Braunschweig, Schleinitzstraße 20, D-38106 Braunschweig, Germany; 2 Department of Vaccinology and Applied Microbiology, EM-Unit, Helmholtz Centre for Infection Research, Inhoffenstraße 7, D-38124 Braunschweig, Germany; 3 Fiber and Polymer Technology, KTH Royal Institute of Technology, Teknikringen 56-58, SE-10044 Stockholm, Sweden

**Keywords:** cyanoethyldextran, cyanoethylpullulan, ferromagnetic nanoparticles, glycan nanostructures, substitution pattern

## Abstract

Cyanoethylglucans with a degree of substitution in the range of 0.74 to 2.40 for dextran and 0.84 to 2.42 for pullulan were obtained by Michael addition of acrylonitrile to the glucans under various conditions. Products were thoroughly characterized, comprising elementary analysis, NMR and ATR–IR spectroscopy, and analysis of the substituent distribution in the glucosyl units by GC–FID and GC–MS of the constituting monosaccharide derivatives. Nanostructuring of the highly substituted cyanoethylpolysaccharides was performed by dialysis against a non-solvent. In the presence of ferromagnetic iron-oxide nanoparticles, multicore cyanoethylglucan-coated ferromagnetic nanoparticles were formed by selective entrapment. The specific interaction between cyano groups and iron could be proven. The size distribution and morphology of the nanoparticles were analyzed by dynamic light scattering (DLS), scanning electron microscopy (SEM) and energy-filtered transmission electron microscopy (EF–TEM) with parallel electron energy loss spectroscopy (PEELS).

## Introduction

Cyanoethylation has been widely applied to polysaccharides, e.g., to cellulose [1], inulin [2], and starch [3]. Onda reported on cyanoethylation of pullulan with degrees of substitution (DS) up to 2.71 [4]. In contrast to Williamson-type etherifications, the base is not consumed in this nucleophilic addition of acrylonitrile, which is a reversible and thermodynamically controlled reaction. While *O*-cyanoalkylglycans are of interest as such, they have also been used as precursors for amino-functionalized polysaccharides [2,5–8].

Introducing a cyanoethyl group offers various potential advantages. First, cyanoethylpolysaccharides show remarkable electric properties: These compounds used as gel electrolytes exhibited an enhanced ionic conductivity up to 2.4 × 10^−3^ S/cm. Thus, a lithium ion polymer battery with cyanoethylpullulan as a matrix polymer could be built with high charge/discharge efficiency [9]. Another example is a vertical electrochemical transistor based on poly(3-hexylthiophene), which was realized by making use of the film-forming qualities of cyanoethylpullulan [10].

Partial hydrophobization of polysaccharides by the introduction of nonpolar residues enables nanostructuring by self-assembly of these compounds. Heinze et al. demonstrated that hydrophobic dextran derivatives form spherical particles on the nanometer scale when a solution of the polymer material is dialyzed against the poorer solvent water. A certain degree of hydrophobicity and amphiphilic balance is necessary to form stable particles [11–14]. We also observed nanostructure formation of alkynyldextrans [15]. Embedding magnetic iron cores in the polymer particle allows for control by magnetic fields. Magnetic separation techniques or magnetic particle imaging can be performed. Applications, such as drug delivery or targeting, hyperthermia and biosensing can be realized [16–18]. Binding of stabilizing organic shells to ferric oxide nanoparticles is usually mediated by carboxylate groups [19]. The interaction of magnetic nanoparticles, coated with glucans (cellulose, pullulan and dextran), with human cells was reported by Heinze et al. [17,20].

A prerequisite for any application in pharmaceutical as well as technological fields is the structural characterization of the material. Cyanoethylation is established for polysaccharides, but the substituent pattern has only been studied for cyanoethylamylose and starch [3]. In most cases, the products have only been roughly characterized by NMR and IR spectroscopy or by elementary analysis [1–2]. Verraest described the substituent distribution in *O*-cyanoethylinulin by HPLC analysis and ^13^C NMR spectroscopy [7]. The structure and the solution properties of cyanoethylcellulose were investigated by FT–IR and ^13^C NMR spectroscopy, as well as by light scattering [21–22]. Cellulose and starch derivatives have been studied more extensively due to their frequent use, e.g., market share and bulk flow in industrial processing [23].

**Figure 1:**
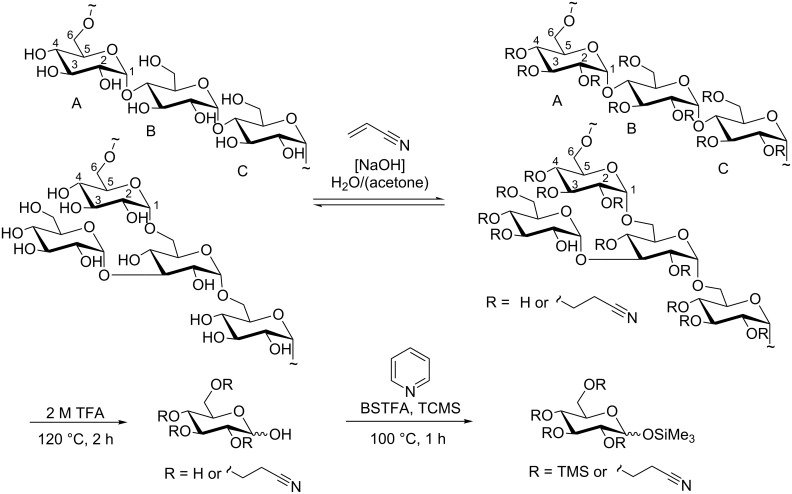
Polysaccharide structures of pullulan and dextran cyanoethylation with acrylonitrile and NaOH as catalyst; sample preparation for GLC/GLC–MS analysis: depolymerization and silylation.

Pullulans and dextrans (Figure 1) have not received the attention these fascinating polymers deserve, due to their lower trading volume and higher price. But in recent years their importance for special biochemical or pharmaceutical applications, such drug delivery or biosensor technology, has grown, due to their special properties such as water solubility, low viscosity and film formation [24–27]. Pullulan is a homopolysaccharide of D-glucose secreted by *Aureobasidium pullulans*. The repeating units of this linear and regular glucan are maltotrioses (Glc-α-1→4-Glc-α-1→4-Glc), which are α-1→6-linked. Due to this linkage pattern, pullulan is very flexible and dissolves readily in water, with low viscosity. Films can be easily prepared. Pullulan is nontoxic, even edible, biocompatible and biodegradable [28–29]. Several applications in pharmaceutical and food technology have been reported. A summary for biotechnological applications of pullulan is given by Leathers [30].

Dextran is also a microbial glucan, e.g., from *Leuconostoc mesenteroides*. The main chain is α-1→6-linked and, in contrast to pullulan, randomly branched to various extent at positions O-3, O-4 and/or O-2, beside short stumps consisting of 1 to 2 glucose units; the 1–2% long-chain branching influences the properties of commercially available dextran [31–33]. Due to their nontoxicity and biocompatibility, dextrans are applied as blood-plasma expander. Dextran derivatives are used in many biomedical and bioanalytical applications [34] and are the subject of further developments in this field [35]. Therefore, we selected pullulan and dextran as candidates for a linear and a branched polysaccharide in our cyanoethylation studies. Cyanoethylglucans of different DS values were produced. Another objective of our approach was the detailed determination of the substitution pattern on the monomer level. Furthermore, nanostructuring of highly substituted cyanoethylglucans with and without ferromagnetic nanoparticles was investigated.

## Results and Discussion

### Synthesis and characterization of cyanoethylglucans

Dextran (6 kDa) and pullulan (100 kDa) were reacted with different amounts of acrylonitrile (AN) and sodium hydroxide in water. According to the patent of Onda [4], acetone was applied as a solubility mediator in some reactions. Reaction conditions were varied to obtain scarcely, moderately and highly substituted cyanoethylglucans. Products were isolated and purified by dialysis. Reaction parameters are shown in Table 1. DS values ranging from 0.74 to 2.40 (dextran, CED-1–CED-3) and 0.84 to 2.42 (pullulan, CEP-1–CEP-3) were obtained by GLC analysis. Up to a DS of around 1.50, the products were still water-soluble. Derivatives with a DS above 2 showed good solubility in acetone, DMSO or DMF. Product characterization was carried out by elementary analysis (EA), ^1^H NMR spectroscopy, and infrared spectroscopy (ATR–IR). Gas–liquid chromatography (GLC) in combination with mass spectrometry (MS) was employed for the analysis of glucose derivatives after depolymerization of the cyanoethylglucans.

**Table 1:** Conditions and results of cyanoethylation of dextran (6 kDa) and pullulan (100 kDa) with acrylonitrile (AN) and NaOH.

sample			CED-1	CED-2	CED-3	CEP-1	CEP-2	CEP-3

reaction	mass educt	[g]	1.20	1.20	0.50	1.20	1.20	0.50
conditions		[mmol/glc]	7.4	7.4	3.1	7.4	7.4	3.1
	H_2_O	[mL]	4	4	5	4	4	5
	acetone	[mL]	–	1	4.75	–	1	4.75
	NaOH	[equiv/glc]	0.2	0.2	2	0.2	0.2	2
	AN	[mL]	1.9	1.9	4.65	1.9	1.9	4.65
		[equiv/glc]	4	4	23	4	4	23
	time	[h]	0.5	0.5	24	0.5	0.5	24
	temperature	[°C]	45	45	20	45	45	20
	mass product	[g]	1.23	1.30	0.77	1.17	1.29	0.71

DS	DS_EA(N)_^a^		0.86	1.46	2.68	0.90	1.35	2.32
	DS_EA(CN)_^b^		0.91	1.55	2.72	0.97	1.42	2.32
	DS_NMR(1)_^c^		1.01	1.81	2.37	–	–	–
	part. DS_NMR(2)_ at O-2^d^		0.47 (46.1)	0.72 (39.6)	–	–	–	–
	DS_NMR(3)_^e^		0.97	1.61	2.51	0.89	1.31	2.43
	DS_GC_^f^		0.74	1.39	2.40	0.84	1.52	2.42
	DS_GC_^g^		0.85	1.52	2.52	0.91	1.55	2.48

yield^h^			77–83	86–74	82–86	74–76	72–75	79–81

^a^DS calculated from elementary analysis (based on N content). ^b^DS calculated from elementary analysis (based on C/N ratio). ^c^DS calculated from ^1^H NMR according to Equation 1. ^d^Partial DS in position 2 calculated from ^1^H NMR according to Equation 2, % of total DS_NMR(1)_. ^e^DS calculated from ^1^H NMR according to Equation 3. ^f^DS calculated from GLC analysis; only cyanoethyl derivatives considered. ^g^DS calculated from GLC analysis; *O*-carboxyethyl side products (TMS esters) are included. ^h^Range calculated based on the lowest and highest DS value obtained by the different methods a–c, e and f.

**Figure 2:**
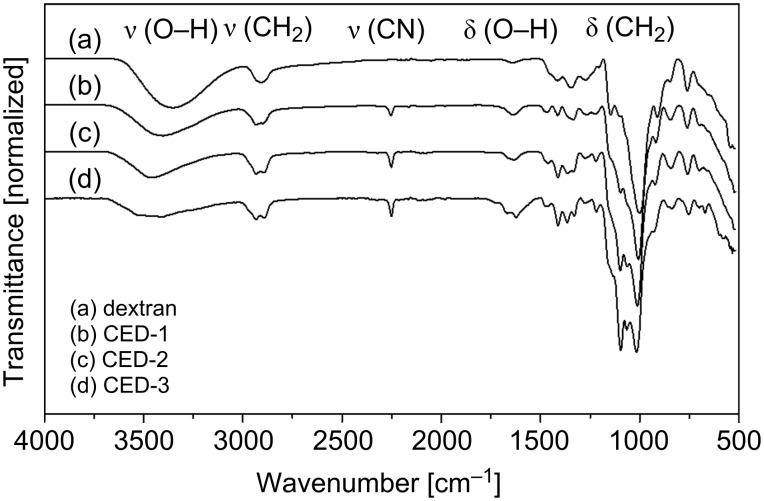
ATR–IR spectra of (a) dextran, 6 kDa, and cyanoethyldextrans (b–d) CED-1–3.

**Figure 3:**
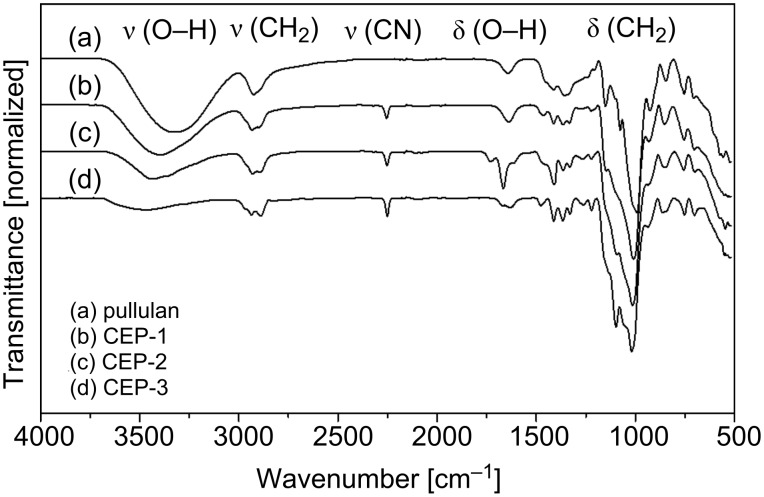
ATR–IR spectra of (a) pullulan, 100 kDa, and cyanoethylpullulans (b–d) CEP-1–3.

The DS of heterogenic atoms containing polysaccharide derivatives can be followed by elementary analysis. To avoid misinterpretations due to impurities of the polymer sample, it should be checked whether the DS usually calculated from the nitrogen content is in accordance with the ratios of the other elements. Therefore, we evaluated the DS_EA_ from the N content and additionally from the C/N ratio. Results are given in Table 1. DS_EA(C/N)_ was 0 to 7.8% higher than DS_EA(N)_. In the following the functionalized glucans were investigated by ATR–IR spectroscopy. The characteristic C≡N stretching vibration was detected at 2250 cm^−1^ increasing with DS, while the intensity of the OH stretching (3400 cm^−1^) decreased, with the maximum being shifted to higher wavenumbers (→ less hydrogen bonding). No side products, such as amides or carboxylates (as hydrolysis products of nitrile groups), or only traces thereof, were observed [21]. ATR–IR spectra of native dextran and the cyanoethyl ethers are shown in Figure 2. Figure 3 shows the ATR–IR spectra for pullulan and the corresponding cyanoethyl ethers.

**Figure 4:**
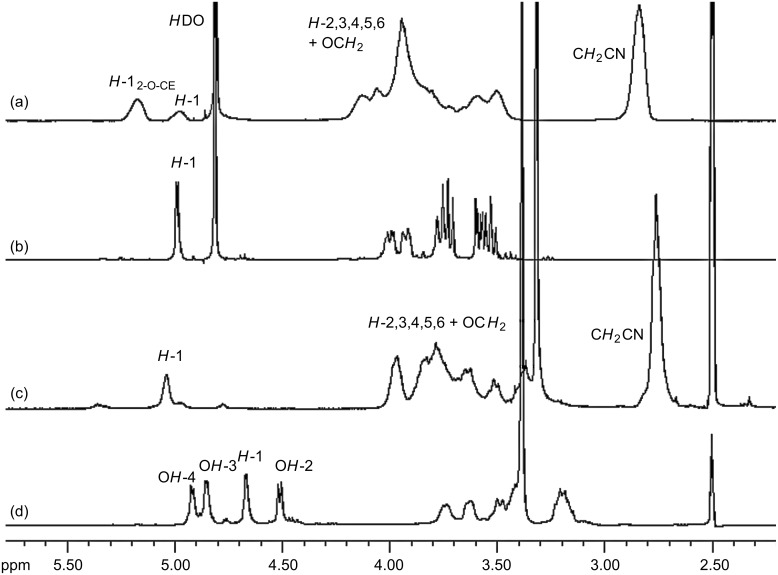
^1^H NMR spectra (300 MHz) of (a) CED-2 (DS_NMR(1)_ = 1.81) in D_2_O; (b) dextran, native in D_2_O; (c) CED-3 (DS_NMR(1)_ = 2.37) in DMSO-*d*_6_; (d) dextran, native in DMSO-*d*_6_; calibrated with solvent signals.

^1^H NMR spectroscopy is a versatile and fast method for qualitative and quantitative structural analysis. Figure 4 presents the ^1^H NMR spectra of cyanoethyldextrans CED-2 and CED-3 in comparison with the unmodified polysaccharide. DMSO-*d*_6_ was used as a solvent for the derivatives with a DS > 2 and D_2_O for the less-substituted polyglucans.

**Figure 5:**
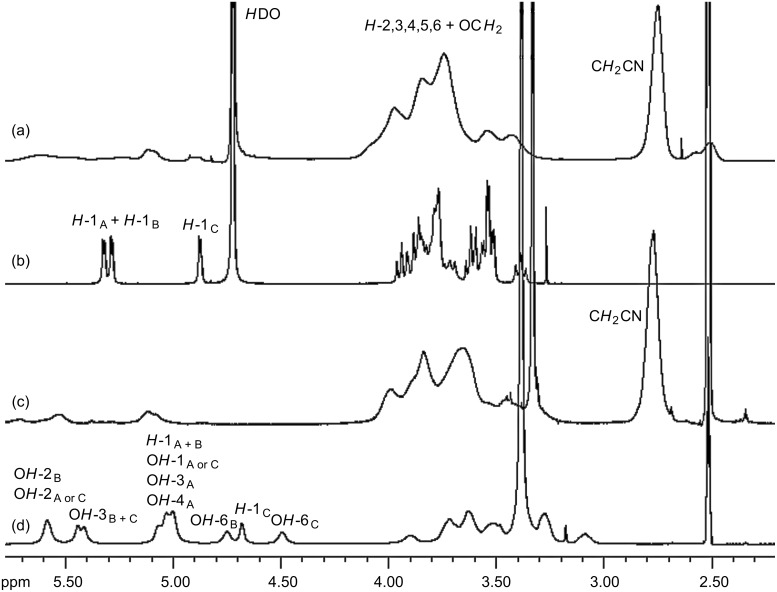
^1^H NMR spectra (400 MHz) of (a) CEP-2 (DS_NMR(3)_ = 1.31) in D_2_O; (b) pullulan, native in D_2_O; (c) CEP-3 (DS_NMR(3)_ = 2.43) in DMSO-*d*_6_; (d) pullulan, native in DMSO-*d*_6_; calibrated with solvent signals.

The spectra of *O*-cyanoethylglucans show strong peak broadening and therefore worse resolution compared to the starting material (Figure 4 and Figure 5). The signal at 2.82 ppm in D_2_O and 2.75 ppm in DMSO is assigned to the methylene group adjacent to the cyano function (C*H_2_*–CN). The remaining protons of the cyanoethyl substituent overlap with sugar ring protons in the range of 3.3–4.2 ppm. As a result of 2-*O*-substitution, *H*-1 is shifted downfield. For the α-1→6-glucosyl residues of dextrans (in D_2_O) it is shifted from 4.97 to 5.16 ppm. The average DS value was calculated from the ratio of the signal integrals of the methylene group adjacent to the nitrile group, to the summarized integrals of *H*-1 (Equation 1). DS evaluation in position 2 is also possible (Equation 2).

[1]
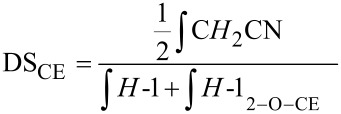


[2]
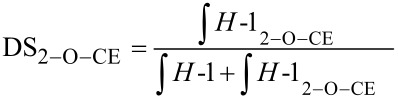


For cyanoethylated pullulan, the situation is more complex as is obvious from Figure 5. While the NMR spectrum of the native pullulan in D_2_O is well resolved with *H*-1 signals at 4.90 (α-1→6-Glc, ring C), 5.30 ppm (α-1→4-Glc, ring A) (α-1→4-Glc, ring B), the signals are shifted downfield by *O*-cyanoethylation and peak broadening occurs, probably due to a poorer solution state of the much more hydrophobic derivatives, and higher viscosity. The region of the anomeric protons becomes very complex and is difficult to integrate. It is assumed that for the 2*-O*-cyanoethylglucoses, the *H*-1 of the glucosyl residues A, B and C (Figure 1) are differentiated by ^1^H NMR spectroscopy. In DMSO the OH resonances overlap with *H*-1 protons (Figure 5c and Figure 5d) [36]. The signals are shifted by substitution and the resolution becomes poor. Nevertheless, DS can alternatively be estimated from the integral of the CE-methylene group at 2.82 and the sugar ring protons, which are corrected for the equal contribution by the CE substituent according to Equation 3.

[3]
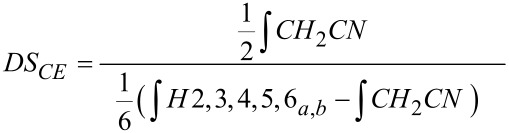


**Figure 6:**
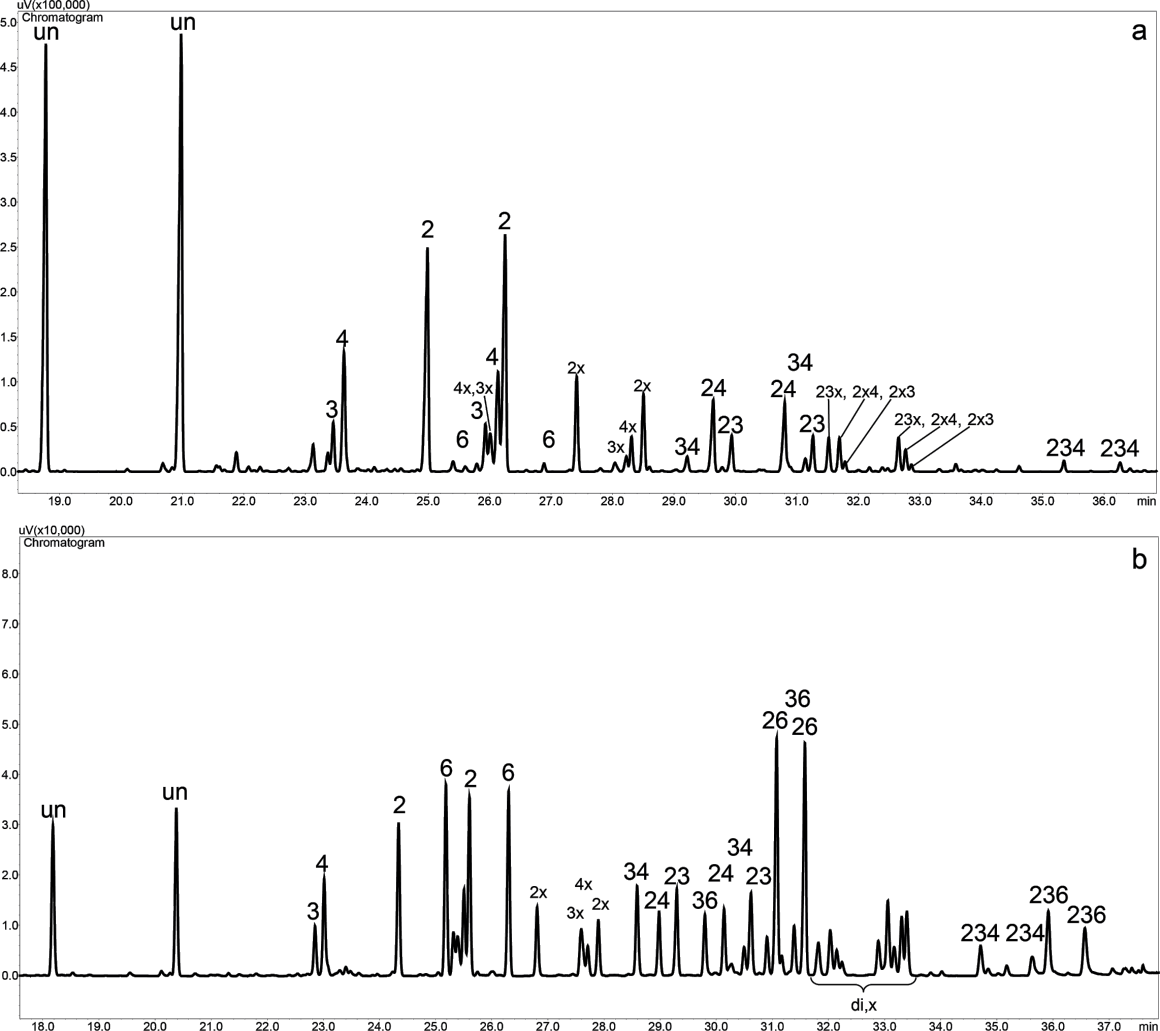
Gas chromatogram of hydrolyzed and trimethylsilylated cyanoethylglucans; (a) CED-1 (DS_GC_ = 0.74); (b) CEP-2 (DS_GC_ = 1.52); x = side products with hydrolyzed cyanonitrile, i.e., carboxyethyl-TMS-ester group; di,x = 3x,6CE, 2 or 4x,CE, 2x,6CE, 3x,6CE, 2 or 4x,CE, 2x,6CE); un: unsubstituted glc.

To gain more detailed insight into the distribution of substituents in the glucosyl units, the cyanoethylated glucans were hydrolyzed and subsequently trimethylsilylated. The resulting trimethylsilyl *O*-cyanoethyl-*O*-trimethylsilyl-α,β-D-glucosides were analyzed by GLC–FID [3]. Peaks were assigned according to the position of cyanoethylation by GC–MS (Figure 6). Pairs of α- and β-glucosides were observed for each pattern. In addition, minor peaks of *O*-carboxyethyl derviatives (as SiMe_3_ esters) were observed, since hydrolysis of the cyano group could not be avoided completely. However, quantitative evaluation, with and without considering these side products, did not effect significant differences in the relative substituent distribution, but only an underestimation of the average DS_GC_ of up to 12%. Obviously, the rate of hydrolysis of the peripheral CN groups is decoupled from the carbohydrate backbone and thus independent of the position. Both DS_GC_ values are given in Table 1.

Based on monomer analysis, a statistical evaluation can be performed. Neglecting 6-*O*-substitution at terminal residues, eight different constituents are expected for cyanoethyldextran: unsubstituted (*s*_0_), monosubstituted at position 2, 3, or 4 (*s*_2_, *s*_3, _*s_4_*), disubstituted at positions 2,3, 2,4, or 3,4 (*s*_23_*, s*_24,_* s*_3_*_4_*), and 2,3,4-tri-*O*-substituted glucose (*s*_234_). For pullulan, 6-, 2,6-, 3,6-, and 2,3,6-patterns must be additionally considered. With the exception of 6-*O*-CE-glc, these patterns were not detected for cyanoethyldextrans. The tetra-*O*-substituted glucosyl unit (*s*_2346_), a possible product of the terminal residue, was neither detected for pullulan nor for dextran ethers.

For the statistical evaluation of the monomer data of cyanoethyldextrans, substitution at O-6 was neglected, i.e., 6,*n*-*O*-substituted glucosyl units were added to the *n*-*O*-substituted group. The number of monomer patterns considered was thus reduced to eight. In case of pullulan, consisting of α-1→6 linked maltotriose repeating units (Figure 1), patterns including 4- or 6-*O*-substitution were weighted according to their availability of 1:2 calculated for a random distribution. Random patterns were calculated considering the partial DS values determined for the different OH groups. The results of monomer analysis and statistical evaluation are summarized in Table 2. The peak areas from GLC–FID measurements were corrected according to the effective response concept [37].

**Table 2:** Monomer composition [mol %] (*s*_n_) of un-, mono-, di- and tri-substituted glucose units (*c_n_*) (DS_GC_ calculated without *O*-carboxyethyl derviatives) and partial degrees of substitution (*x_n_*) at position 2, 3, 4, 6; *H*_1_: heterogeneity parameter.

		CED-1	CED-2	CED-3	CEP-1	CEP-2	CEP-3

monomer	*s*_0_	43.20	14.97	2.29	37.90	11.32	0.49
composition	*s*_2_	24.61	21.76	2.92	25.39	11.82	1.05
[mol %]	*s*_3_	4.85	5.39	2.65	4.28	3.52	0.88
	*s*_4_	11.86	12.19	3.38	3.65	6.86	1.26
	*s*_6_				9.02	13.98	6.63
	*s*_23_	4.02	11.41	13.58	2.94	7.03	3.69
	*s*_24_	8.46	21.36	6.80	2.97	5.23	1.12
	*s*_26_				6.28	19.66	15.07
	*s*_34_	1.80	4.46	15.39	4.20	5.26	6.54
	*s*_36_				1.62	4.81	10.14
	*s*_234_	1.20	8.46	52.99	0.33	3.34	16.53
	*s*_236_				1.41	7.17	36.60

number of	*c*_0_	43.20	14.97	2.29	37.90	11.32	0.49
cyanoethyl groups	*c*_1_	41.31	39.34	8.95	42.35	36.17	9.82
	*c*_2_	14.28	37.23	35.78	18.01	41.99	36.55
	*c*_3_	1.20	8.46	52.99	1.74	10.51	53.14

partial DS values	*x*_2_	0.38	0.63	0.76	0.39	0.54	0.74
	%	52.12	45.27	31.85	47.05	35.76	30.56
	*x*_3_	0.12	0.30	0.85	0.15	0.31	0.74
	%	16.15	21.35	35.34	17.69	20.52	30.70
	*x*_4_	0.23	0.46	0.79	0.11	0.21	0.25
	%	31.73	33.38	32.81	13.35	13.64	10.50
	*x*_6_				0.18	0.46	0.68
	%				21.92	30.07	28.24

	**DS**	**0.74**	**1.39**	**2.40**	**0.84**	**1.52**	**2.42**
	*H*_1_^a^	0.30	0.39	0.51	0.43	0.43	0.55

^a^
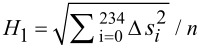
, ∆*s*_i_ = *s*_i_ (theor.) – *s*_i_, with *s*_i_ = mol fraction of glucose units substituted in position i, *n* = 8 for dextran, *n* = 12 for pullulan; (mean values calculated by twofold determination of the molar composition of the corresponding TMS derivatives by GLC–FID).

CED-1 and CEP-1, modified without the cosolvent acetone, showed the lowest DS values. Upon addition of 1 mL acetone (Table 1), while the other parameters of the reaction were maintained, the DS value increased by nearly 100% (CED-2 and CEP-2). The highest DS values were achieved under the conditions according to Onda (CED-3 and CEP-3) [4]. In this approach acetone and acrylonitrile were used in the same ratio. The base concentration and reaction time were increased (2 equiv NaOH/glc) and the reaction was performed at rt. Acetone acts as a solvent intermediator and probably improves the contact of acrylonitrile and glucan. In addition, it keeps the product in a solution state, even at increasing DS.

**Figure 7:**
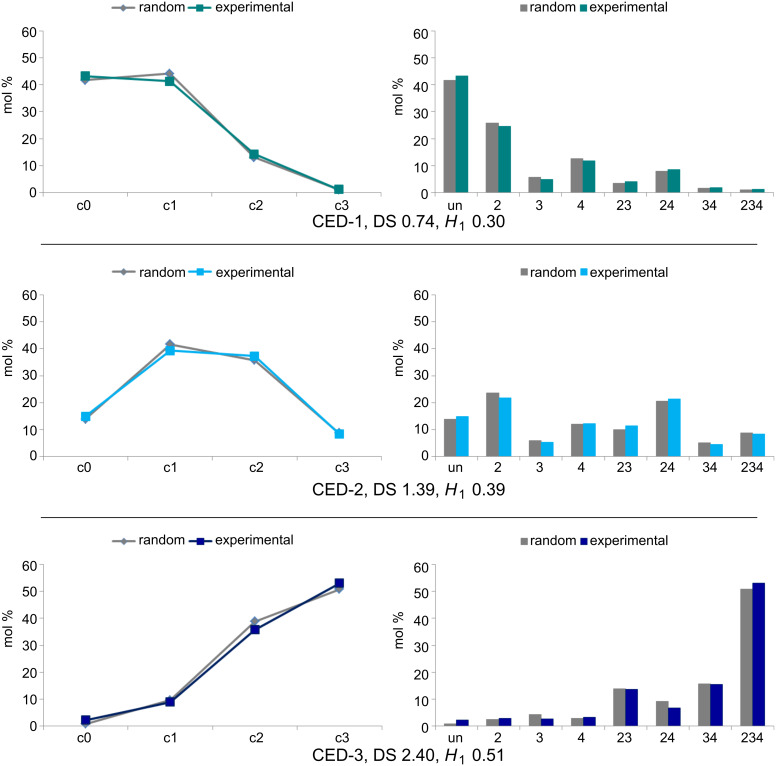
Experimentally determined substituent distribution in the glucosyl units (glc) of cyanoethyldextrans CED-1–3 (blue) compared with a random distribution (gray). Left: ci = the fraction of i-fold-substituted glucosyl units (mol %); right: un = unsubstituted glc; the numbers assign the substituted positions, e.g., 234 = 2,3,4-tri-*O*-cyanoethyl glc; *H*_1_ heterogeneity parameter for the standard deviation as defined in Table 2.

**Figure 8:**
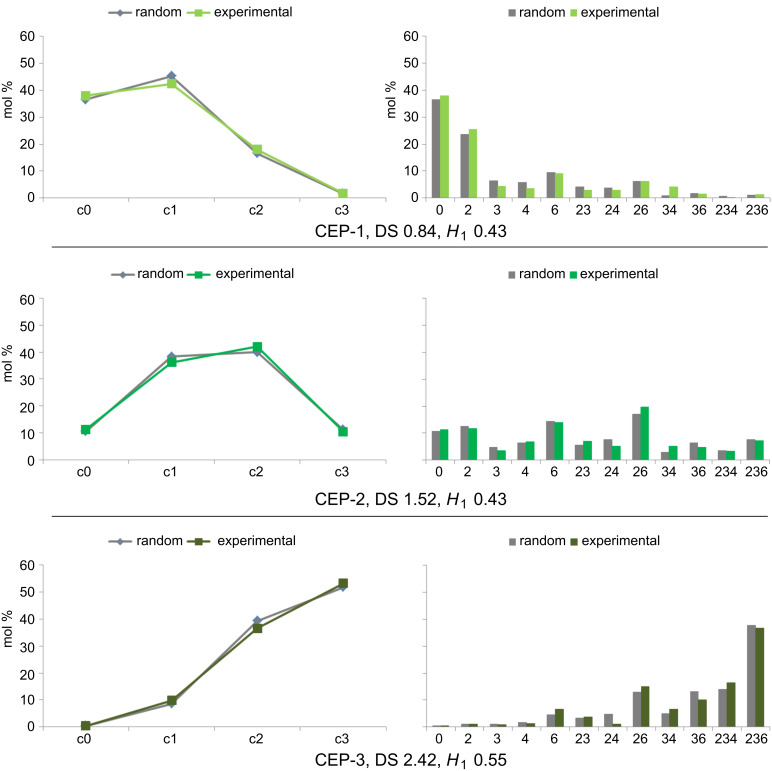
Experimentally determined substituent distribution in the glucosyl units (glc) of cyanoethylpullulans CEP-1–3 (green) compared to a random pattern (gray). Left: c_i_ = the fraction of i-fold-substituted glucosyl units (mol %); right: un = unsubstituted glc; the numbers assign the substituted positions, e.g., 234 = 2,3,4-tri-*O*-cyanoethyl glc; *H*_1_ heterogeneity parameter for the standard deviation as defined in Table 2.

The results of GLC analysis demonstrate that the thermodynamically controlled regioselectivity for the cyanoethylation follows the order O-2 > O-4 > O-3 for all CE-dextrans. The order of partial DS values for CE-pullulans changes with increasing DS (and thus with the reaction conditions), in favor of the primary 6-OH. Considering the relative proportions of the OH groups, namely 3:3:1:2 for O-2, O-3, O-4, and O-6, the degree of conversion follows the order O-6 > O-4 > O-2 > O-3 for CEP-2 (DS = 1.52), with the three secondary OH being equalized for CEP-3 (DS = 2.42). Only for CEP-1, with the lowest DS (0.84), the most acidic 2-OH dominates, and 4-OH shows higher conversion than primary O-6. Comparing pullulan with dextran, the preference for 2-*O*-cyanoethylation is less pronounced in pullulans, in which one third of the 4-OH is “substituted” by the primary 6-OH. In former work, we found a higher preference for 6-O-substitution (50%) over 2-O-substitution (37%) for exclusively α-1→4-linked amyloses reacted in an aqueous paste [3]. The heterogeneity parameter *H*_1_ indicates the average deviation of experimental data from a random distribution, taking into account the relative partial DS values (*x*_i_) found for the various OH groups. A DS dependency of *H*_1_ is inherent when employing this equation, since DS is limited to the range of 0–3. The highest heterogeneity can be calculated at medium DS values of around 1.5. Approximating the limits (DS = 0 and 3), less deviation is possible. The evaluated heterogeneity values are low, as was expected for a thermodynamically controlled reaction [38]. Minor deviations from the random model are within experimental error. Corresponding graphics are shown in Figure 7 and Figure 8.

By all three of the methods applied, similar DS values were obtained. Elementary analysis is a fast method employing the entire material. However, it is important not only to take the nitrogen content for the estimation of DS, but also to check whether the relative amounts of C and H are in accordance with this, since the N content can be lowered by nitrogen-free impurities or be enhanced by residual side products from the reagent. DS_GC_ values calculated by including the *O*-carboxyethyl derivatives were in good agreement with the DS_EA_. The DS_GC_ calculated values without these side products were decreased. This is plausible, since the side reaction mentioned makes the product pattern even more complex and does not allow detection and identification of all minor components, thus discriminating the DS. NMR spectroscopy can be applied to the intact polymer and gives more detailed information than EA. However, broadening and splitting of peaks into different types of *H*_1_ depending on the position of linkage and substitution makes it difficult to assign and integrate all relevant resonance signals. This is obvious from the differences obtained when employing different signals for the calculation (Equation 1 and Equation 3). Only after depolymerization it was possible to determine the detailed substitution pattern by GLC analysis.

In general, good isolated yields in the range from 72 to 86% were achieved (Table 1). The DS values strongly increased upon the addition of acetone as a solubility mediator (CED-2 and CEP-2). No side products, such as amides or carboxylates, or only traces thereof, were detectable for the CE glucans, as proved by IR and NMR measurements (^1^H and ^13^C). A homopolymerization of acrylonitrile could be excluded since the DS_EA_ from N and C/N were very close and only moderately enhanced compared to the DS_GC_.

### Nanostructures of cyanoethylglucans

**Figure 9:**
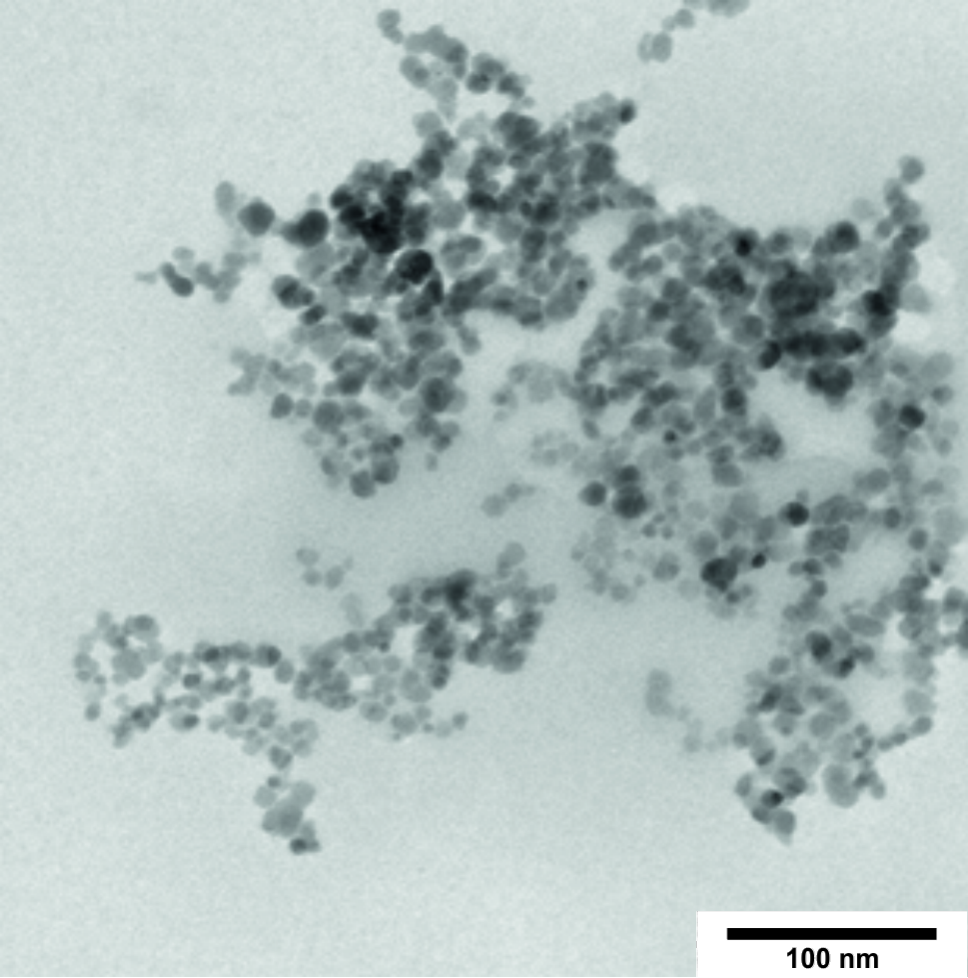
TEM micrograph of iron oxide nanoparticles prepared from an aqueous dispersion.

In the next step the ability of cyanoethylglucans to form nanoparticles was investigated [12–14,39]. DMSO solutions of the cyanoethylpolysaccharides CEP-3 (DS_GC_ = 2.42) and CED-3 (DS_GC_ = 2.40) were submitted to dialysis against water. Only the derivatives with a DS value >2 formed regular particles that were stable in water for several weeks without precipitation [13]. Furthermore, the same procedure was performed in the presence of ferromagnetic iron oxide nanoparticles. The magnetic cores were prepared by a precipitation process of Fe(II) and Fe(III) chlorides (molar ratio 1.7:1.0) with aqueous ammonia solution [40–41]. After ultrasonic treatment the resulting particles were fixed with strong magnets and washed with distilled water. Monodisperse and regularly shaped iron oxide nanoparticles were obtained as shown by TEM (Figure 9). The iron concentration of the nanoparticle dispersion, as analyzed with inductively coupled plasma optical emission spectroscopy (ICP–OES), was 37.2 g/kg. The particle size was calculated by image data processing of the TEM micrograph resulting in a mean diameter of 12.2 nm ± 2.6 nm. The hydrodynamic diameter was estimated at 27 nm by dynamic light scattering (DLS) measurements. Agglomeration or aggregation processes were prohibited by pH stabilization. At pH 2 the iron oxide dispersion is stable for several months without precipitation.

After the dialysis process of the high-DS cyanoethyldextran and pullulan (20 mg, CED-3, DS_GC_ = 2.40; CEP-3, DS_GC_ = 2.42) the hydrodynamic diameter of the particles was determined by DLS. Additionally, scanning electron microscopy (SEM) micrographs were recorded. Using SEM the morphology of polysaccharide particles is accessible. The parameters and results of the DLS and SEM measurements are summarized in Table 3.

**Table 3:** Parameters and characterization of nanostructures formed from CED-3 and CEP-3, in the absence and presence of ferromagnetic nanoparticles, by DLS and SEM measurements; 20 mg of cyanoethylglucan was used for each entry (= 0.07 mmol glucosyl units).

sample	CE-glucan^a^	iron oxide nanoparticle dispersion		diameter		
		[μL]	Fe [mmol]	DLS	SEM ± ^b^	
				[nm]	[nm]	No^c^

1	CEP-3	10	0.0067	611	613 ± 174	(69)
2	CEP-3	20	0.0133	399	388 ± 93	(335)
3	CED-3	10	0.0067	337	514 ± 205	(92)
4	CED-3	20	0.0133	444	451 ± 113	(27)
5	CED-3	200	0.1332	252	–	
6	CEP-3	–	–	241	260 ± 57	(182)
7	CED-3	–	–	203	331 ± 71	(308)

^a^20 mg (= 0.07 mmol glucosyl units, or 0.165 mmol CE); ^b^standard deviation; ^c^number of evaluated particles

**Figure 10:**
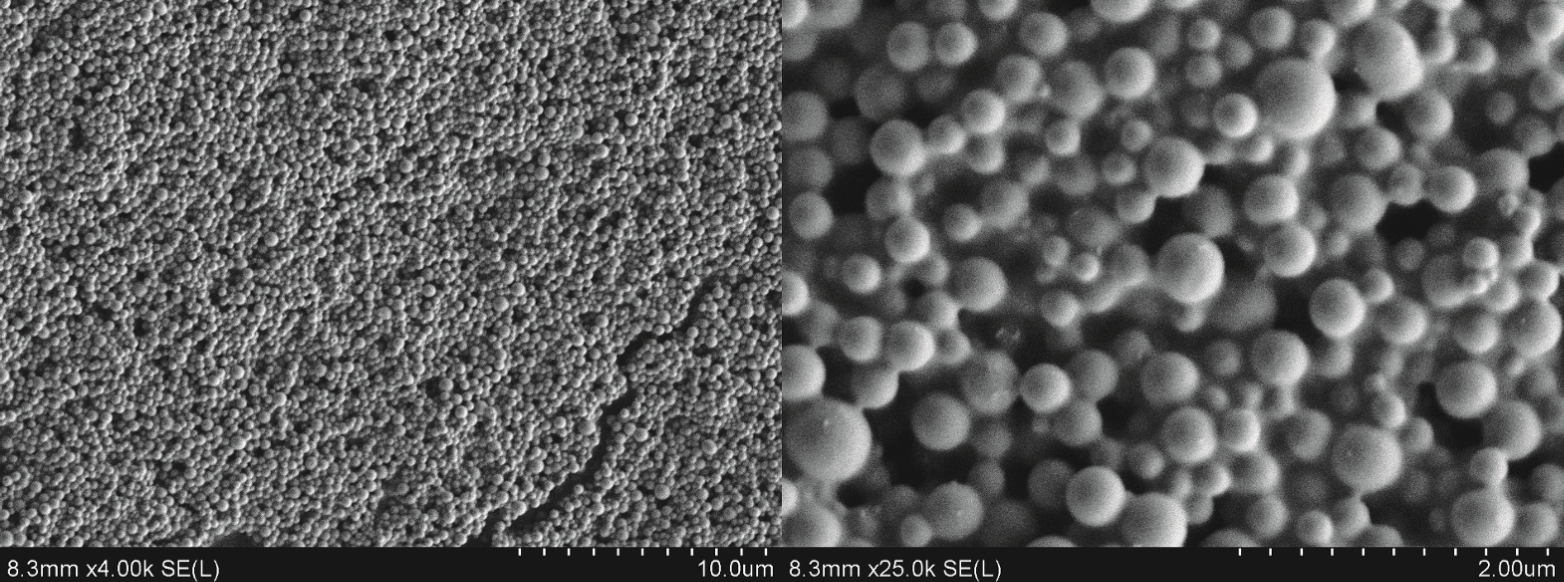
SEM micrographs of CEP-3 with iron oxide nanoparticles, (Table 3, entry 2).

DLS measurements were in good agreement with the evaluation of the electron microscopy images. According to the micrographs, the morphology of the cyanoethyl nanoparticles can be considered as spherical. Representative SEM pictures of CEP-3 + Fe-np (Table 3, entry 2) are shown in Figure 10.

The influence of different amounts of ferromagnetic nanoparticle dispersion was investigated (10–200 μL or equivalently 0.0067–0.1332 mmol Fe/0.07 mmol glucosyl units, or 0.165 mmol CE). Independent preparation of the ferromagnetic nanoparticles has the advantage that nanostructuring can be performed on a small scale. Coprecipitation methods, in which magnetic particles are formed and simultaneously coated, require higher amounts of polymer [42] (up to gram scale) and deliver irregularly shaped particles [13]. Increasing the amount of metal oxide from 0.0133 to 0.1332 mmol iron (20–200 μL) with 20 mg polymer (= 0.07 mmol glucosyl units, respective 0.165 mmol CE) resulted in smaller particles (Table 3). The upper limit is 200 μL ferrofluid (0.1332 mmol Fe/0.165 mmol CE). Below this limit the whole of the iron core is entrapped. Higher amounts of iron oxide resulted in multimodal, aggregated particles and precipitation. Interestingly, the smallest particles were formed without iron oxide (Table 3, entry 6 and 7, 260 nm, respectively 331 nm). Magnetic properties depend on the amount of iron and can be adjusted by varying the doping of the glucan particles with iron. Nanostructuring of cyanoethyldextrans and cyanoethylpullulans show no significant differences, although in one case a branched polymer with 6 kDa and on the other hand a 100 kDa linear macromolecule was employed.

**Figure 11:**
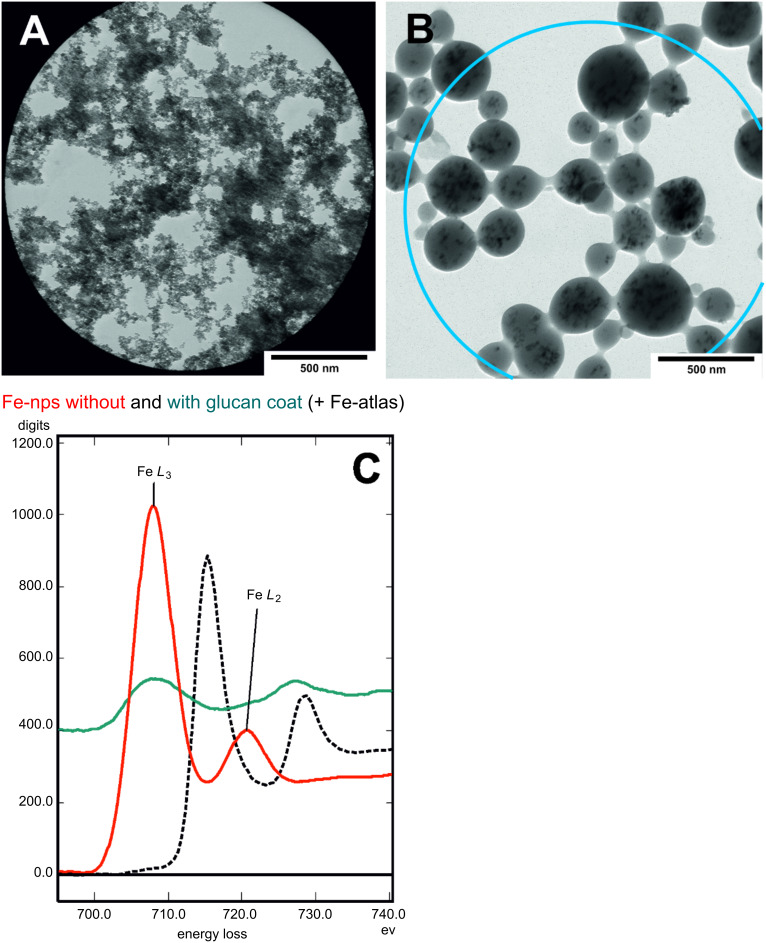
TEM micrograph of (a) uncoated iron oxide nanoparticles; (b) of CEP-3 + iron oxide nanoparticles (no stain), Table 3, entry 1; (c) PEELS measurements of uncoated iron oxide particles (red line), polysaccharide coated particles (blue line) and the Fe(0) atlas reference spectrum (black, dashed line); PEELS measuring areas are shown as aperture in A and circle in B; for details see text.

**Figure 12:**
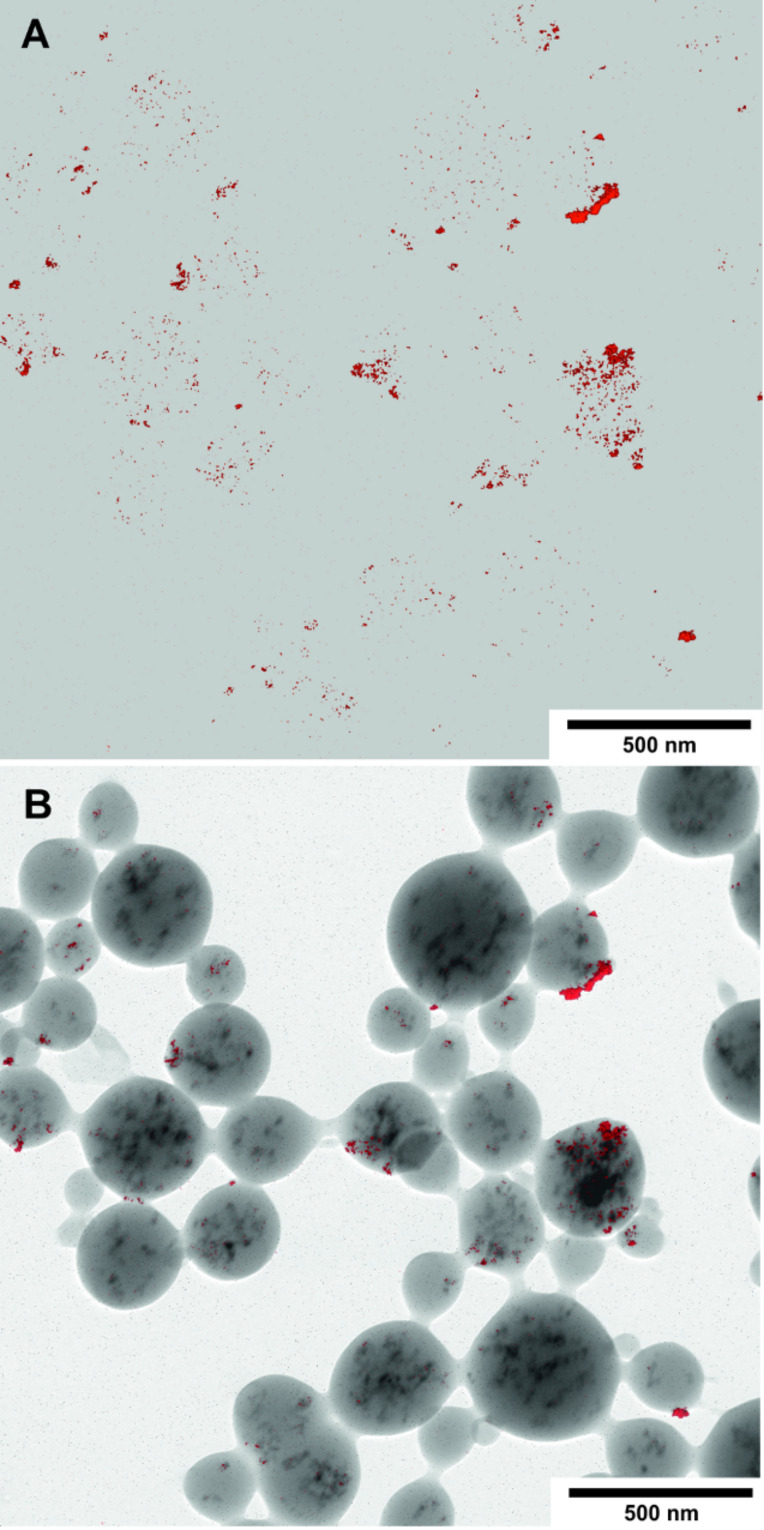
ESI Fe distribution maps of CEP-3 with iron oxide nanoparticle (Table 3, entry 1). (A) Net Fe, shown in red. Because of the signal dimensions, much of the signal intensity is lost, leading to only a weak mapping signal within the corresponding particles; (B) Fe map (red) overlaid on the zero-loss image.

Energy-filtered transmission electron microscopy (EF–TEM) is an appropriate method to characterize the structure, morphology and the redox state of metal-containing nanoparticles. Parallel electron energy loss spectroscopy (PEELS) analyses were performed with the uncoated and coated iron oxide nanoparticles in Figure 11. The energy loss functions have been summed from 690 to 740 eV. Figure 11c shows the Fe *L*_2,3_ edge spectra of the uncoated iron oxide particles (red line) relative to the polysaccharide coated particles (blue line) and a reference iron, Fe(0) spectrum (dashed black line) [43]. The intensity is comparably low (Figure 12c) due to the particle size, exceeding the ideal thickness of 30 to 40 nm for EELS analysis. Nevertheless, a typical intensity profile of Fe *L*_2,3_ can be seen, at an energy resolution of 1.2 eV. The uncoated metal oxide particles show maxima of 708.2 eV and 720.7 eV for the *L*_3_ respective *L_2_* edge. In the case of the coated particles, the maxima were determined at 711.4 eV for *L*_3_ and 728.9 eV for the L_2_ edge, showing a significant chemical shift, and differences in the spin orbit splitting, i.e., 17.5 eV for coated iron oxide cores versus 12.5 eV for noncoated particles were observed, based on individual measurements. The *L*_3_–*L*_2_ peak maxima distance is defined as the spin orbit splitting for “white-line” elements, such as transition elements and lanthanides, and the *L*_3_/*L*_2_ ratio is indicative of the oxidation state of the element [44–45]. Cressey described the possibility to characterize multiple valence states of 3*d* metals by *L*-edge spectra [46]. By comparing our data with theirs, it is shown that Fe^2+^ and Fe^3+^ are present. The spin orbit splitting reflects the influence of the electronic state of the iron oxide core and obviously an interaction with the nitrile groups should be considered [45,47].

Entrapping of iron oxide cores during the carbohydrate nanostructuring process is proven by the electron micrographs. Figure 12 shows the net iron distribution, colored red, in the multicore particles in detail. Due to the material thickness, which is beyond the ideal 30–40 nm, common for EELS measurements, only iron oxide particles near the surface show strong intensity signals. No free iron particles were detected on the carbon foil of the electron microscopic grid or in the waste water after the dialysis step, proving that all of the iron was specifically bound by the cyanoethylglucans. The hydrophobic cyanoethyl groups are expected to “hide” inside the particles, but depending on the distribution of these residues, some can also be directed towards the water phase. These substituents may bind iron oxide particles additionally. This observation could indicate that some cyanoethyl groups are available for further transformation of the outer sugar shell, e.g., amino functionalization followed by coupling with bioactive molecules. In conclusion, it was shown, that the magnetic iron cores were captured by the cyanoethyl-functionalized polysaccharides.

## Conclusion

Cyanoethylation by Michael addition is a versatile tool for polysaccharide modification. The hydrophobic substituents were introduced up to a DS of ca. 2.4 through choice of the appropriate conditions. Average reactivity of the α-glucans dextran and pullulan was very similar. The order of substitution was O-2 > O-4 > O-3 for dextran, while the relative degree of conversion changed with the DS from O-2 > O-4 > O-6 > O-3, in favor of primary O-6 for pullulan. The substituents present are randomly distributed in the glucosyl units, which is typical for reversible reactions and always favored in aqueous systems. High cyanoethylated glucans form regularly shaped nanostructures with diameters in the range of 260 to 613 nm. When dialysis was performed in the presence of ferromagnetic nanoparticles, glucan-coated multicore ferromagnetic nanostructures were formed. Quantitative entrapment of iron oxide during dialysis is obviously based on interactions of the cyanoethyl residues with the iron oxide core particles, as is indicated by TEM and PEELS measurements. Further modification of the cyanoethylglucans and their respective nanostructures by transformation to aminopropyl derivatives is under progress. These new particles possess great potential as precursors for amino-functionalized, magnetic architectures and electrochemical applications.

## Experimental

### Materials

Dextran from *Leuconostoc ssp*. (6 kDa) and pullulan (100 kDa) were purchased from Fluka. Acrylonitrile (AN) was supplied from Janssen. DMSO [puriss, absolute, over molecular sieves (H_2_O ≤ 0.01%), ≥ 99.5% (GC)] was obtained from Sigma-Aldrich. Deionized water was used. Dialysis was performed with molecular porous dialysis membranes (molecular weight cut off 3.5 kDa) from Spectrum Laboratories. Bidistilled water was chosen for ICP–OES sample preparation.

### Instrumentation

^1^H NMR spectra were acquired on a Bruker AMX 300 spectrometer or a Bruker AMX 400 MHz Advance spectrometer at rt (around 5 mg sample in D_2_O or DMSO-*d*_6_). Chemical shifts are given in ppm relative to the residual solvent signals. ATR–IR spectra were recorded by using a Bruker Tensor 27 attenuated total reflectance infrared (ATR–IR) spectrometer. Elementary analysis (EA) was performed on a Thermoquest EA 1112 analyser. The data given is always the average of two measurements.

Gas–liquid chromatography (GLC) analysis was carried out with a GLC–FID instrument Shimadzu GC 2010 with a Phenomenex Zebron ZB5-MS column (30 m, i.d. 0.25 mm, ﬁlm thickness 0.25 mm and 1.5 m). H_2_ (40 cm s^−1^, linear velocity mode) was used as a carrier gas. Data were recorded with a Shimadzu GC Solution Chromatography Data System (version 2.3). Peaks were identified by gas chromatography/mass spectrometry (GLC–MS) analysis. Conditions: injector 250 °C, temperature program: 60 °C (1 min); 20 °C/min to 130 °C, 4 °C/min to 260 °C, 50 °C/min to 310 °C (10 min), splitless. GLC–MS: Agilent 6890 GC (ZB5-MS column, 30 m, inner diameter 0.25 mm, ﬁlm thickness 0.25 mm and 1.5 m) and a JEOL GC mate II bench-top double-focusing magnetic sector mass spectrometer. The iron content was determined with a Radialen ICP–OEC Vista MPX, from Varian, (power 1.20 kW, plasma gas 15 L/min (Ar), auxiliary gas 1.5 L/min (Ar), atomizer pressure 240 kPa, pump speed 20 rps). Atom emission lines: ion emission lines: 234.350/238.204/239.563/259.940/260.709/261.187 nm, and internal reference line: Ar, 470.067 nm. Sample preparation: 0.408 g iron nanoparticle dispersion was dissolved by adding 5 mL HCl (37%) and subsequently diluted to 100 mL with bidistilled water (ultrapure). Calibration was carried out with an external standard solution: Fe 10.000 mg/L (Specpure, Fa. Alfa Aesar).

### Particle size determination

The hydrodynamic size was determined by using a Zetasizer Ver 6.0.1, Malvern Instruments Ltd. Scanning electron micrographs were recorded with a Hitachi S-4800 FE-SEM (Tokyo, Japan) at KTH, Sweden. Samples were prepared by putting a drop of each sample on the carbon film covered the metal studs and allowing it to dry. Samples were kept for at least 48 h in the desiccator prior to the analysis. Transmission electron micrographs were obtained by using a EF–TEM Libra 120 plus Zeiss microscope operated at 120 kV. The samples were adsorbed to a hydrophilized carbonﬁlm, which was supported by a Cu grid (carbon only, copper 300 square mesh) and dried at rt. All images, PEELS and ESI-sets were recorded with a 2×2k SharpEye cooled CCD camera (Tröndle, Moorenweiss, Germany) and directed by the ITEM software (OSIS, Münster, Germany). PEELS spectra (main settings: emission current = 2 µA; spectrum magnification 100×; illumination aperture = 0.5 mrad; spectrum registration = 5 s;) and ESI series (magnification 12500×; spectrometer entrance aperture = 100 µm; slit width = 9 eV; illumination aperture = 0.8 mrad; image registration = 50 s; E_max_: 712 eV; W1: 690 eV; W2: 660 eV; emission current: 2 µA) of Fe and oxygen were processed, following adaptively the workflow as described by Hedrich et al. [48].

### Cyanoethylation

#### Samples with DS < 2

The glucan (1200 mg, 7.4 mmol glc 100 kDa pullulan or 6 kDa dextran) was dissolved in water, and NaOH (60 mg, 0,2 equiv/glc) and acrylonitrile (1,941 μL, 4 equiv/glc) were added. In syntheses of CED-2 and CEP-2 1 mL, acetone was added. The mixture was stirred at 45 °C for 30 min. The product was purified from low-molecular-weight reagents and by-products by dialysis and freeze dried.

#### Samples with DS > 2

According to Onda [4], acrylonitrile (4.65 mL, 23 equiv/glc) and acetone (4.75 mL) were added to a solution of the polysaccharide (500 mg, 3.08 mmol glc 100 kDa pullulan or 6 kDa dextran) in water and NaOH (250 mg, 2 equiv/glc). The solution was stirred for 24 h at rt. The product was dissolved by the addition of acetone and precipitated by adding water three times.

Elementary analysis: CED-1: C, 47.65; H, 6.12; N, 5.79; CED-2: C, 50.06; H, 5.82; N, 8.53; CED-3: C, 54.60; H, 5.96; N, 12.23; CEP-1: C, 47.08; H, 6.27; N, 5.99; CEP-2: C, 50.10; H, 6.02, N 8.10; CEP-3: C, 54.55; H, 6.23; N, 11.41.

IR (diamant–ATR): 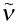 = 3465 (s, OH, depending on DS), 2923, 2893 (m, CH, CH_2_, aliph.), 2252 (w, C≡N, nitrile), 1640 (m, OH), 1167, 1467, 1409, 1324, 1271 (m, CH), 1099, 1009 (s, C-O).

^1^H NMR of CED-1 and CED-2: ^1^H NMR (D_2_O, 300 MHz) δ (ppm) 5.19 (1H, *H*-1,substituted at position 2), 5.00 (1H, *H*-1, unsubstituted), 4.20–3.37 (6H + DS × 2H, *H*-2,3,4,5,6a,b + OC*H*_2_), 2.82 (2H, C*H*_2_CN); CED-3: ^1^H NMR (DMSO-*d*_6_, 400 MHz) δ (ppm) 5.03 (1H, *H*-1), 5.40–4.64 O*H*-4, O*H*-3, O*H*-2 overlapped by *H*-1 shifted due to substitution), 4.10–3.62 (6H + DS × 2H, *H*-2,3,4,5,6,ab + OC*H*_2_), 2.76 (2H, C*H*_2_CN).

^1^H NMR of CEP-1 and CEP-2: ^1^H NMR (D_2_O, 300 MHz) δ (ppm) 5.77–4.86 (O*H*-2, O*H*-3, of glucosyl unit A, B and C + *H*-1, substituted and unsubstituted of glucosyl unit A, B and C), 4.21–3.36 (6H, H-2,3,4,5,6,a,b + OC*H*_2_), 2.80 (2H, CH_2_CN); CEP-3: ^1^H NMR (DMSO-*d*_6_, 400 MHz) δ (ppm) 5.95–4.75 (O*H*-2, O*H*-3, of glucosyl unit A, B and C + *H*-1, substituted and unsubstituted, glucosyl unit A,B and C), 4.11–3.20 (6H, *H*-2,3,4,5,6,a,b + OC*H*_2_), 2.77 (2H, C*H*_2_CN).

### Monomer analysis of cyanoethylpolysaccharides

The monomer composition of cyanoethyl derivatives was determined by GLC (twofold determination) after hydrolysis and trimethylsilylation according to [3]. The effective carbon response concept was applied for quantitative GLC–FID evaluation [37]. Peak areas were corrected by multiplication with the following factors: TMS O-CE/O-TMS-Glc: un- ≡ 1.0000, mono- 1.0694, di- 1.1491, and trisubstituted 1.2416; carboxyethyl-TMS-ester (from hydrolysis of the nitrile group): mono- 0.9250, di- 0.9840 (CH_2_CH_2_COOTMS/CE), 0.8605 (2 × CH_2_CH_2_COOTMS), trisubstituted 1.0511 (2 × CH_2_CH_2_COOTMS/CE).

### Preparation of nanoscaled structures

#### Iron nanoparticle dispersion

A solution of a mixture of Fe(II) and Fe(III) chlorides in a molar ratio of 1.7:1.0 was prepared in distilled water. Aqueous ammonia solution (25 mL, 25%) was added and the mixture was heated at 70 °C for 30 min. After ultrasonic treatment the resulting particles were fixed with strong magnets, and the nonmagnetic products were removed by washing with distilled water three times and adjusted to pH 2.1 with HCl [17,40–41].

#### Polysaccharide nanoparticles

Polysaccharide (20 mg) was dissolved in 5 mL DMSO. Nanoparticles of cyanoethylglucans were prepared by a dialysis process against water [12]. For entrapping of iron oxide nanoparticles, various portions were mixed with the cyanoethylpolysaccharides before dialysis.
